# Integrating DRN-RF with computer vision for detection of control room operator’s mental fatigue

**DOI:** 10.1371/journal.pone.0320780

**Published:** 2025-04-09

**Authors:** Zuzhen Ji, Xian Xie, Enjing Jiang, Yuchen Wang, Bohan Min, Shuanghua Yang, Yong Chen, Dirk Pons

**Affiliations:** 1 Department of Mechanical Engineering, Zhejiang University of Technology, Hangzhou, Zhejiang Providence, China; 2 Department of Chemical Engineering and Biological Engineering, Zhejiang University, Hangzhou, Zhejiang Providence, China; 3 Department of Mechanical Engineering, University of Canterbury, Christchurch, Canterbury, New Zealand; Sri Krishna College of Engineering and Technology, INDIA

## Abstract

Control room operators encounter a substantial risk of mental fatigue, which can reduce their human reliability by diminishing concentration and responsiveness, leading to unsafe operations. There is value in detection of individuals’ mental fatigue status in the workplace. This study introduces a new method for mental fatigue detection (MFD) that combines computer vision and machine learning. Traditional methods for MFD typically rely on multi-dimensional data for fatigue analysis and detection, which can be challenging to apply in a real situation. The traditional methods such as the use of biological data, e.g., electrocardiograms, require operators to be in constant contact with sensors, while this study utilizes computer vision to collect facial data, and a machine learning model to assess fatigue states. The developed machine learning method consists both Deep Residual Network and Random Forest (DRN-RF). A comparison with existing MFD methods, including K Nearest Neighbors and Gradient Boosting Machine, has been carried out. The results show that the accuracy of the DRN-RF model reaches 94.2% and the deviation is 0.004. Evidently, the DRN-RF model demonstrates high accuracy and stability. Overall, the proposed method has the potential to contribute to improving the safety of process system operations, particularly in the aspect of human factor management.

## 1. Introduction

Avoidance of mental fatigue plays a significant role in ensuring safe operations [1–4]. The primary factors leading to mental fatigue include insufficient sleep, inadequate periods of rest, disruptions to circadian rhythms, and high levels of stress [5–7]. Common manifestations of mental fatigue include reduced concentration and responsiveness, which can subsequently lead to slips, lapses, and mistakes [8], or diminishes an individual’s capacity to understand [9] and react [10], which may contribute to incidents [11–13]. Operators are particularly susceptible to mental fatigue due to the repetitive nature of their tasks, the substantial mental workload involved, and prolonged computer usage [14]. The specific area under examination here are control room operators (CROs) in chemical industry. These workers typically operate from centralized command centers and are responsible for production process control.

Traditional approaches to assess mental fatigue often rely on subjective measures and self-assessment tools, such as the Stanford Sleepiness Scale (SSS) [15], Karolinska Sleepiness Scale (KSS) [16], and Fatigue Assessment Scale (FAS) [17,18]. These assessments can be influenced by analysts’ personal biases. Objective assessment methods, on the other hand, are developed based on biological, physical, and operational data [19]. These objective methods have found applications in fields like vessel operation [20] and vehicle driving [21]. Despite the advantages of objective methods, challenges persist. First, data collection for biological signals such as electrocardiogram (ECG), electromyogram (EMG), electrooculogram (EOG), and electroencephalogram (EEG) requires physical contact between operators and sensors [22,23]. However, applying such equipment, especially EEG and ECG, in production work sites is challenging and may disrupt the normal activities of CROs. Second, the accuracy of these assessments heavily relies on the quality of the biological data. In many industrial settings, biological signals are susceptible to interference from noise, leading to less reliable results. Third, mental fatigue detection (MFD) is a real-time process, and existing methods require CROs to wear bio-sensors continuously, which is often impractical in real-world scenarios. These factors collectively reduce the feasibility of implementing traditional MFD methods in practical industrial applications [24].

To address the aforementioned issues, this paper introduces a new method for assessing the mental fatigue of control room operators in the chemical industry, which combines computer vision and machine learning techniques. In contrast to existing MFD methods which relying on biological data like ECG and EEG [25], our approach utilizes computer vision to gather facial data from operators, which can be easily captured by cameras. This innovation significantly enhances the practical applicability of the MFD method. In the process of collecting data such as ECG and EEG, it is typically necessary to utilize precision sensors that require being worn by individuals. However, in the context of the daily work environment, maintaining the proper functioning and continuous use of such sensors over an extended period poses significant challenges. Conversely, the method proposed within this research enables data collection merely through the camera of a computer. This approach eliminates any potential interference with the actual work carried out by the operator, thereby substantially enhancing its practicality.

Moreover, an innovative model integrating the Deep Residual Network (DRN) with the Random Forest (RF), namely the DRN-RF model, has been developed specifically for the purpose of Monitoring Fatigue Degree (MFD). When contrasted with traditional approaches like the K-Nearest Neighbors (KNN) and the Gradient Boosting Machine (GBM), the DRN-RF model demonstrates remarkable advantages. It not only achieves a higher level of accuracy in detecting visual fatigue but also significantly bolsters the stability of the monitoring process. This is examined by a comparative experiment in this paper. This novel combination and its outstanding performance thus represent a significant leap forward in the realm of visual fatigue monitoring methodologies.

The article is structured as follows. Section 2 discusses the existing methods for MFD. Section 3 introduces the overall MFD framework and the development of DRN-RF model. Section 4 presents a pilot study to validate the proposed method and conducts comparative research to demonstrate its superiority over other existing MFD methods. Section 5 addresses the main contribution of the work, limitations, and conclusion.

## 2. Literature on mental fatigue detection

### 2.1. Existing mental fatigue detection methods

Detection methods have demonstrated significant utility in assessing human fatigue. These methods find extensive application across various industries, including nuclear power plants [26] and transportation [27]. The evolution of mental fatigue assessment can be traced back to the 1950s when studies initially employed techniques like “Blink Counting” for fatigue detection [28]. Human mental fatigue detection techniques can be classified into three categories: mathematical models; rule-based models; and machine learning models. Mathematical models monitor human fatigue by considering long-term data inputs, such as circadian cycles, duration of sleep, duration of wakefulness, and sleep history [19]. The Two Process Model [29] and Three Process Model [30] represent the earliest mathematical models and are widely utilized in industries that regulate working hours, such as railway transportation and aviation [31]. However, the approach evaluates human fatigue over prolonged durations, which may lack precision.

Rule-based models classify input and output states, making them a simplified implementation of an expert system. Fuzzy Inference Systems (FIS) provides a preferred decision-making method that integrating fuzzy membership functions and fuzzy rules. The IF-THEN rule is employed to establish a mapping from input to output. For example, in Devi and Bajaj’s research [32], the states of eyes are divided into blink, sleep, and slept, while the states of the mouth are divided into yawning and normal. Features including mouth states and eye states are input into a two-layer FIS, and the output is categorized as fit, fatigued, or dangerous [33]. This type of model can be trained even in the absence of data and possesses the capacity to acquire knowledge from additional knowledge bases. A limitation of rule-based models is that they only consider partial features related to mental fatigue, such as mouth and eye states. However, these selected partial features may not fully capture the complexity of mental fatigue. Consequently, the effectiveness and reliability of detection technology may be compromised.

Machine learning models include shallow models and deep learning models. Shallow models require training data and preprocessed features to provide reasonable results. Different shallow models are used in fatigue detection, including Support Vector Machines (SVM) [34,35], eXtreme Gradient Boosting (XGBoost) [20], KNN [36], Decision Tree [37,38], RF [39] and Artificial Neural Networks (ANN) [40–42]. For instance, Friedrichs incorporated external features such as road conditions, road bumps, and cross-wind into an ANN for driving fatigue detection, classifying driver states as awake, fatigued, or questionable [43]. Arun Kumar deploys a novel scheme to predict the driver behavior using five advanced machine learning techniques namely, Logistic regression, Multilayer perceptron, Decision tree, random forest and Naïve bayes algorithm [44]. Liang employed an SVM method to detect factors that may lead to driving cognitive distraction [45]. Comparing with shallow models, deep learning models possess the capability to extract features directly from training data. Convolutional neural networks (CNN) [46–48] are the earliest deep learning models used in MFD. Other deep learning models for MFD are Deep Belief Networks (DBN) [49] and Bayesian Networks (BN) [50], which include Static Bayesian Network (SBN) [51] and Dynamic Bayesian Network (DBN) [52,53]. Compared with mathematical models and rule-based models, machine learning models are more flexible and adaptable in MFD. This is attributed to their enhanced capacity to handle complex and dynamic data.

### 2.2. Mental fatigue feature acquisition

In recent years, several studies have concentrated on human mental fatigue and have demonstrated its significant association with unsafe behaviors [54]. For instance, driving fatigue, a specific form of mental fatigue, is recognized as a major contributor to traffic accidents within the transportation area [55–57]. To effectively reduce the occurrence of unsafe behaviors, various mental fatigue assessment methods have been proposed in different fields [58–60]. These methodologies evaluate fatigue states by analyzing fatigue-related attributes, which can be broadly categorized into four types, including subjective fatigue reporting, biological features, physical features, and operational features [19]. Subjective reporting data are obtained through questionnaires such as the KSS. However, subjective fatigue data generally lacks real-time performance [61], thus it is typically employed for fatigue data labeling rather than as the primary basis for fatigue detection [62]. Biological features encompass biological signals originating from different parts of the human body, such as EEG [25,63], ECG [64,65] and EOG [66]. Physical features directly reflect an individual’s tiredness, including blink frequency, nodding frequency, and the percentage of time the eyes are closed (PERCLOS) [67]. Physical features can be divided into three types, including eye state-based features [68,69], mouth state-based features [70] and head state-based features [71]. For instance, head nodding feature which assessed by x, y, and z coordinates of head through conductivity measurements was used for microsleep event detection [72]. Recent studies have incorporated multiple physical features to enhance detection accuracy [73–75]. Operational features consist of parameters from equipment being operated, which can reflect the mental fatigue state of the operator [76]. For instance, in driving fatigue detection, operational features include vehicular characteristics such as pressure changes on the brake and accelerator pedals, load distribution on the driver’s seat, and vehicle speed [77,78]. To improve the accuracy of MFD, many previous studies have focused on the fusion of various features [79–82].

The prior research has certain limitations. A primary constraint is that acquiring most biological features is challenging in the daily work of CROs. This is because collecting features like EEG and ECG necessitates operators to be in constant contact with precision sensors, which is often infeasible. Furthermore, the equipment used for feature collection may disrupt CROs’ operational activities, and the collected data can easily be affected by the working environment. Addressing these limitations, our research employs computer vision technology to capture human facial data as features for assessing mental fatigue. In contrast to the aforementioned feature acquisition processes, computer vision technology can automatically record an operator’s facial attributes while they are engaged in their normal work activities. This approach eliminates the need for operators to carry sensors or equipment and is applicable within daily work scenarios.

## 3. Methodology

### 3.1. Research preamble

Existing studies in MFD consist with multifaceted limitations. Firstly, in the realm of feature selection, the existing methods necessitate the use of hybrid features as inputs to enhance model accuracy. However, a majority of these features are challenging to collect and use within the daily working environment. For example, subjective fatigue reporting data is recorded after a period of time by participants themselves or other experts, consequently, it is challengeable to record this type of data during the work time. The collection of biological features such as ECG and EEG requires participants a long-time attachment with sensors. In the real working scenarios, a long-time attachment of these sensors may affect participants’ normal working activities, which is unapplicable. Although nowadays the wearable sensors are wildly used in monitoring personnel fatigue; however, high-precision sensors tend to be relatively expensive and impractical for routine use in daily operations. Additionally, certain sensors, such as ECG sensors, are vulnerable to various forms of internal and external noise, including respiratory activity, muscle contractions, and electrical interference. Consequently, our proposed method offers a more cost-effective and stable alternative for fatigue diagnosis compared to the use of high-precision sensors. Alternatively, the quality of data may also be affected by the surrounding environment such as electromagnetic interference from nearby electronic devices, electrical noise and physical collision.

Secondly, facial features are another type of physical features. Different from the above-mentioned fatigue features, facial features are relatively easy to be collected and use in daily working scenarios. This is because facial features are collected by camera which comparing to ECG devices are more stable and applicable in industrial working environment. Additionally, the computer vision technologies are widely used in the collection of facial data. However, most computer vision techniques for human mental fatigue assessment are developed using the eyes and mouth features [83], with relatively limited attention given to other facial features. Nonetheless, certain studies in human biology have underscored the significance of various facial features beyond just the eyes and mouth in reflecting an individual’s mental fatigue, such as eyebrow features [84]. Consequently, solely focusing on eyes and mouth-related features in computer vision-based detection may potentially result in inaccurate diagnostic outcomes.

Thirdly, in this research, the collected facial data exhibit a high dimensionality of features and the characteristic of discrete feature labels. During the process of MFD, many conventional models suffer from issues such as low accuracy and inadequate stability. In response to these limitations, this study proposes a novel DRN-RF model designed to achieve efficient and stable detection of mental fatigue. The proposed model significantly enhance detection accuracy and system reliability, thereby providing a more dependable solution for identifying states of mental fatigue.

In this research, an integration of computer vision and machine learning method namely DRN-RF for CROs’ mental fatigue is proposed to solve above limitations. This method is capable of detecting the occurrence of mental fatigue or identifying its potential presence based on the facial images of subjects. By doing so, it can effectively reduce the likelihood of potential unsafe incidents. Although the principal emphasis of this study lies in the chemical industry, the method proposed herein holds the potential for implications that extend well beyond this particular domain and demonstrates applicability across a wider variety of scenarios.

### 3.2. Overall framework

**Fig 1:**
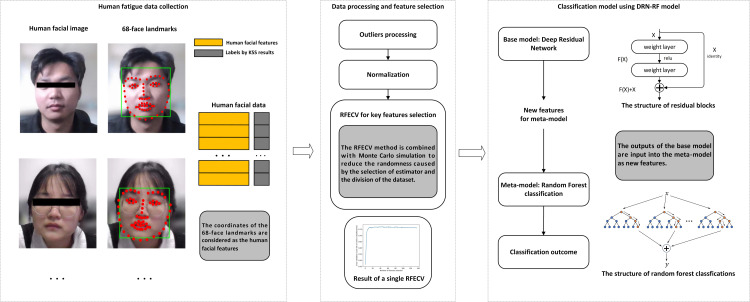
The mental fatigue detection framework.

In this research, we propose an integration of computer vision and machine learning methods for assessing the mental fatigue of CROs. The overall framework is illustrated in Fig 1 (The individual in this paper has given written informed consent (as outlined in PLOS consent form) to publish these case details). The first step involves collecting human facial images using computer vision technology and subsequently extracting facial feature points using a Landmarks algorithm. Following this, the KSS is employed for fatigue labeling. KSS scores are determined through evaluations by two experts and the participant. The final KSS output is obtained as the average of these three evaluations. The second step involves data processing and feature selection. Outliers are initially eliminated, followed by data normalization. In the context of a comprehensive MFD procedure, both data collection and data processing play indispensable roles. The data acquisition phase undertakes the task of transforming the face images captured by the camera into the initial data. Subsequently, through the data processing stage, these data are fed into the DRN-RF classification model as the input data, thereby ultimately obtaining the accurate classification outcomes. The Recursive Feature Elimination with Cross-Validation (RFECV) method is utilized to select facial features relevant to fatigue and eliminate irrelevant ones. To reduce the randomness introduced by the choice of estimator and dataset division, a Monte Carlo process is applied. The third step encompasses fatigue detection using the DRN-RF model. The processed data serves as the input for the base model. Subsequently, the outputs of the base model are considered as new features for the meta model. Finally, the outputs of the meta model yield the definitive classification results for fatigue detection. The proposed method is then applied in a pilot study to validate its effectiveness. Input data information for each particular step in the framework is illustrated in Table 1. To protect the privacy of the participants, all photos were modified to ensure they could not be used for other purposes. The photos and data presented in this paper were approved by the participants. The experiment began on June 7, 2023 and ended on July 8, 2023. Each participant was informed that their facial data and fatigue data would be recorded by photos, and all participants provided their consent through a signed written agreement. Ethical approval for this research was obtained from Zhejiang University, the Committee of College of Biomedical Engineering and Instrument Science, under reference number [2022]-No.48. Mental fatigue dataset can be found in the supporting information section.

**Table 1:** Input data information for each particular step.

Step	Data in put	Feature number	Description
Data collection	Face image	NA	In the pilot study, the face image is taken by 2-seconds intervals, 30mins per person, 10 participants, 7940 valid face images in total.
Data processing	Coordinate information	136	68-lanmarks is used to collect facial coordinate. 68 horizontal coordinates and 68 vertical coordinates are collected per image.
Feature selection	Human mental data	136	Linkage of facial coordinate and KSS. Feature selection using RFECV with Monte Carlo process (100 times).
DRN-RF classification	Selected feature	Determined by Monte Carlo output	Fatigue classification. Training sub-dataset (70% of total dataset); validation sub-dataset (15% of total dataset); test sub-dataset (15% of total dataset).

### 3.3. Data collection and data processing

#### 3.3.1. Face detection using Landmark algorithm.

**Fig 2:**
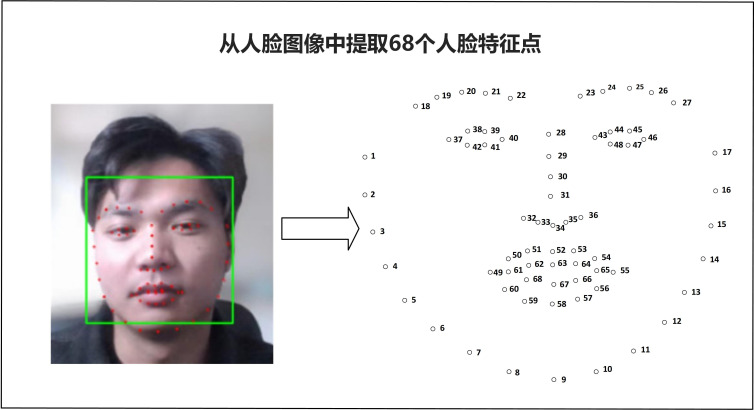
68 special landmarks from face image.

In this research, the Landmark algorithm provided by Dlib is employed for the collection of facial features. The Dlib face alignment algorithm employed in this method would achieves face alignment within milliseconds while maintaining a high level of accuracy [85]. A total of 68 specific landmarks on the face are marked, encompassing points on the contours of the eyes, nose, and mouth, see Fig 2. For human face detection, the Histogram of Oriented Gradient (HOG) feature, combined with linear classifiers, image pyramids, and sliding window detection schemes, is utilized. HOG feature represents a local image feature and is extracted through a series of steps [86]. The first step involves image preprocessing, which includes standardizing the color space using gamma equalization. This step aids in reducing the impact of color variations on feature extraction. The second step entails the calculation of image gradients, where gradient information for each pixel in the image is computed. The third step focuses on generating the gradient histogram. In this phase, the image is divided into small regions referred to as “Cells,” with each Cell containing multiple pixels. Within each Cell, the frequency of gradients in different directions is counted and used to create histograms based on gradient direction and intensity. The fourth step is the normalization step. For each Cell, the histogram is normalized to mitigate the influence of variations in lighting conditions on feature extraction. The fifth step culminates in the generation of the HOG feature. The normalized histograms from all Cells are amalgamated into a single HOG feature vector in this final step.

#### 3.3.2. Human facial data processing.

After data collection, it is necessary to conduct data processing. The first step is outlier processing. In this research, data that do not belong to the range of three sigma above and below the mean value are considered as outliers. The outlier data are deleted and supplemented according to the mean value of the data before and after. The next step is data normalization. Existing methods of data normalization include min-max method, z-score method, and proportional method. In this study, min-max method is selected for data normalization. Z-score method and proportional method are not appliable to our data. The z-score method makes the processed data conform to the standard normal distribution, while the proportional method only applies to sequences where all data are positive. In contrast, the Min-max method performs a linear transformation of the original data, mapping the results to the interval [0,1] (see Equation 1). Therefore, the Min-max method is chosen.


$$n_{p}=\frac{m_{p}-min\text{\{}m_{q}\}}{max\text{\{}m_{q}\text{\}}-min\{m_{q}\}}$$ (1)

Where p and q belong to range [1, l], l represents length of the sequence {m_p_} and{n_p_}, the sequence {m_p_} represents the original sequence, and the sequence {n_p_} represents the converted sequence.

#### 3.3.3. Human facial feature selection.

**Fig 3:**
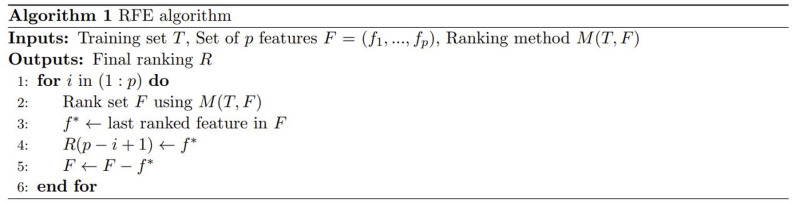
The pseudocode of the RFE algorithm.

The RFECV method is then employed for feature selection. The Recursive Feature Elimination (RFE) method entails fitting a model and iteratively removing features in a recursive manner [87]. In the process of RFE, estimator is an important parameter which affects the feature selection results. Linear Regression evaluators, Decision Tree evaluators, Support Vector Machine evaluators, and Random Forest evaluators are commonly used. Since the integration of human face data and fatigue data is high dimensional and discrete, the Random Forest evaluator is more suitable than other widely used evaluators. Subsequently, features are ranked based on their importance to the model, and the least significant features are systematically eliminated. The pseudocode of the RFE algorithm is indicated in Fig 3 [88].

The Cross-Validation (CV) method is subsequently employed to determine the optimal number of features for the RFE method. Existing CV methods include K-Fold Cross-Validation, Leave One Out Cross-Validation and Leave P Out Cross-Validation. In this RFECV process, K-fold cross-validation is used to score different feature combinations. The K-fold method is used because it is suitable for large sample datasets and, through multiple partitioning, K-fold method reduces the randomness of the results. After cross-validation, the combination with the highest score is selected.

To mitigate the potential randomness introduced by the choice of estimator and dataset division, a Monte Carlo process is implemented. The basic principle of Monte Carlo method is to obtain an approximate solution of the true value by repeated sampling experiments. For each feature, if its occurrence frequency surpasses a certain threshold, the feature is retained; conversely, if it falls below the threshold, the feature is eliminated. In this study, the Monte Carlo RFECV process is repeated 100 times to enhance the reliability of the feature selection process. There are primarily two rationales behind this choice. Firstly, taking into account the constraints of computing resources and computational time, an excessive number of simulations is not advisable. Secondly, upon conducting 100 simulations, it was observed that the results had already reached convergence, rendering further simulations superfluous.

### 3.4. Model development

**Fig 4:**
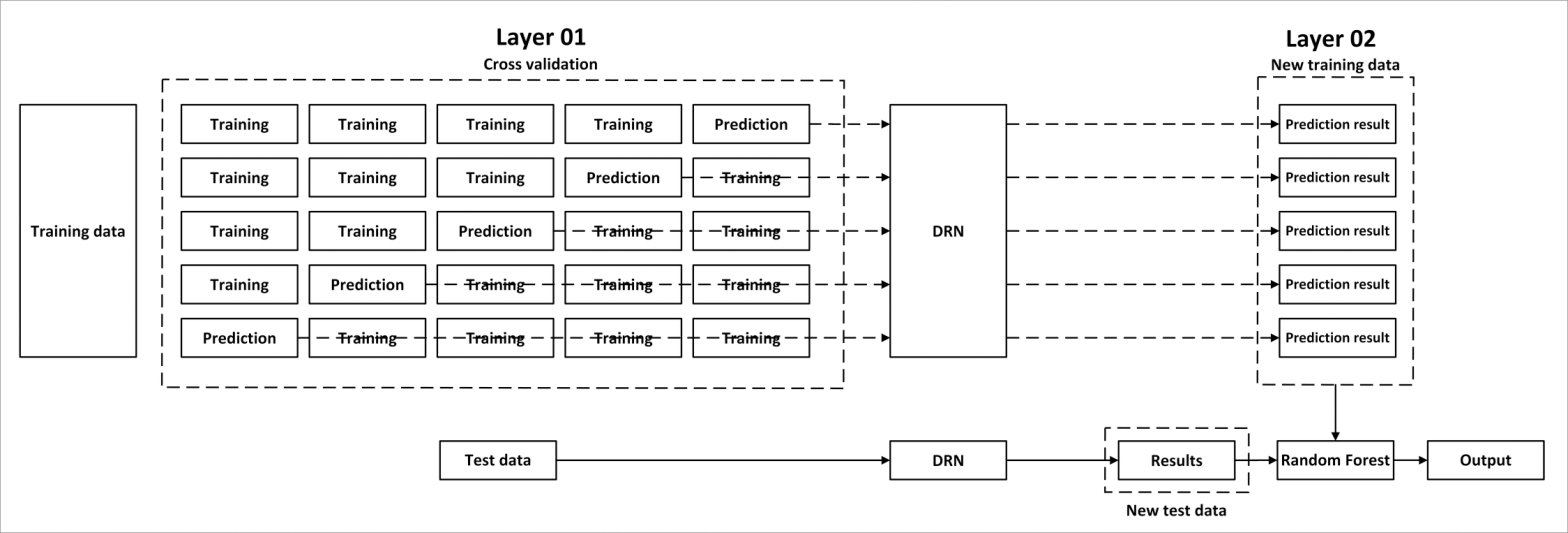
The DRN-RF model.

The DRN-RF model is developed using ensemble learning, combining the advantages of both DRN and RF. The proposed DRN-RF model not only inherits the powerful feature representation capability of DRN, which can effectively address the degradation problem in deep learning, but also exhibits excellent performance in processing high-dimensional data and discrete data as demonstrated by the RF model. The structure of the proposed DRN-RF stacking model is illustrated in Fig 4. In Layer 01, the training data is partitioned into five subsets, with four subsets allocated for training and the remaining subset for prediction. The DRN model in Layer 01 is trained using this K-Fold training method, and the amalgamation of the DRN predictions serves as the new training data for RF classification in Layer 02. The test data is processed similarly, with the predictions generated by the DRN model serving as the new test data for the RF model. The outcome of the RF model constitutes the final classification result.

**Fig 5:**
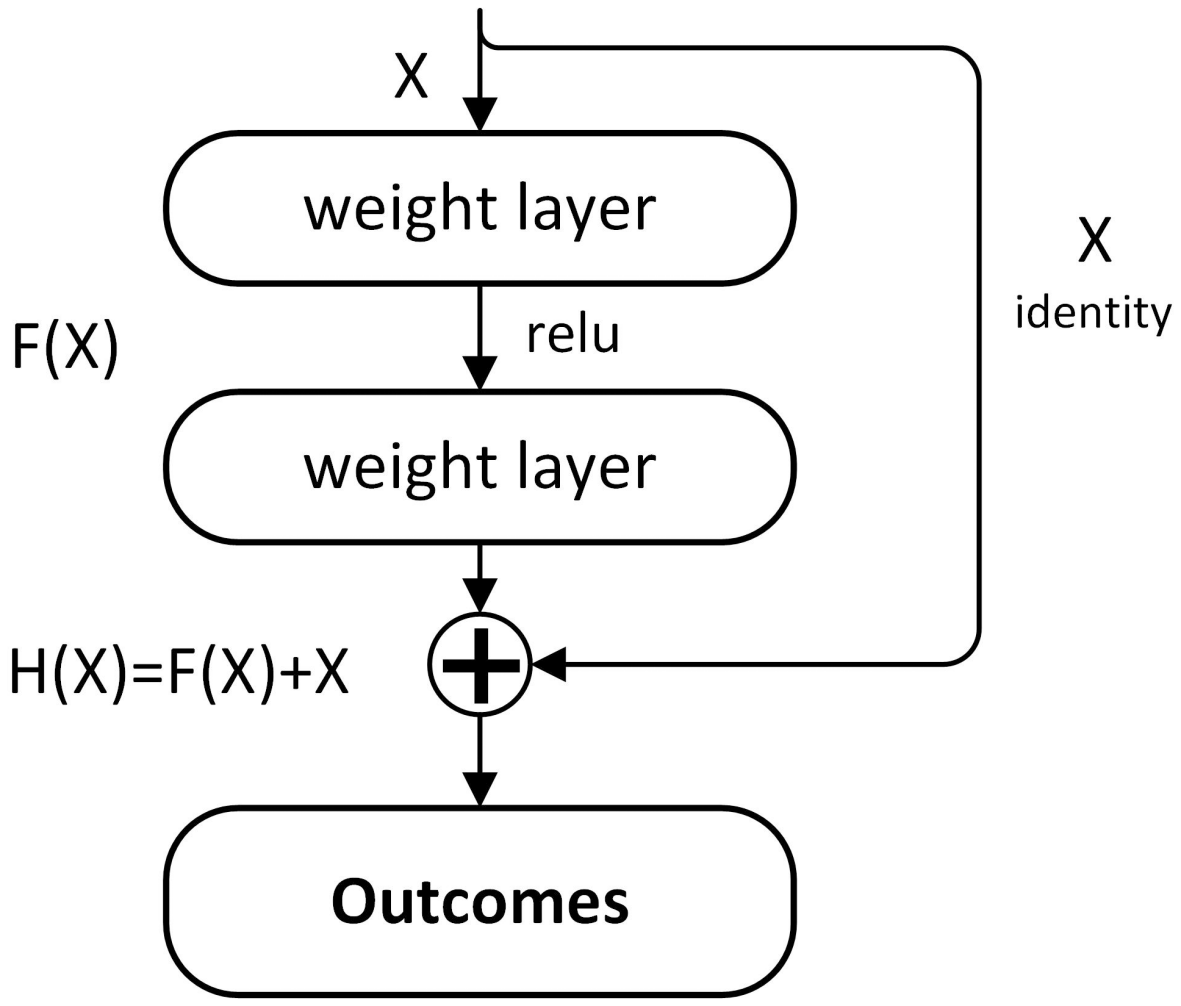
The structure of residual block.

The deep residual network represents a neural network architecture and is a subtype of convolutional neural networks characterized by the presence of “shortcut connections” [80]. These connections utilize the input from the previous layer as the output for one or more subsequent layers positioned below it [89], as shown in Fig 5, where X represents the input, H(X) represents the desired mapping and the shortcut connected weight layers are used to fit the gap between the input and the desired mapping, i.e., *F*(*X*) = *H*(*X*) – *X*.

The depth of deep neural networks is important to machine learning [90]. Recent research shows benefits for visual recognition from very deep models [91,92]. Theoretically, deeper layers of the network tend to exhibit better performance when tackling certain classification problems. However, practical experiments have found that, owing to issues related to vanishing or exploding gradients and the degradation problem, deeper networks often perform worse than shallower networks [93,94]. The problem of vanishing or exploding gradients can be addressed through normalization techniques [88,94,95]. Techniques including Batch Normalization, Dropout, and regularization are incorporated into this model to address issues related to gradient vanishing and explosion. The basic principle of Batch Normalization is to normalize the activation output of the network at each batch of data input. Through this operation, the internal covariance deviation can be greatly reduced, and the learning speed and stability of the model can be improved. Regularization prevent overfitting by reducing the complexity of the model and Dropout is to prevent overfitting by changing the structure of neural network and discarding neurons.

As for the degradation problem, it becomes evident when the deeper layers begin converging, resulting in a rapid decline in model accuracy. This degradation is not primarily caused by overfitting [96], but the problem may be mitigated by a deep residual learning framework [80]. Through the use of shortcut connections, gradients propagate more effectively between layers, enabling the training of ultra-deep neural networks. Consequently, for this MFD study, the DRN model has been chosen for its robust feature representation capabilities. The implementation of shortcut connections also helps preserve the original features (for both on facial and fatigue data), consequently enhancing the accuracy and generalizability of the detection model.

**Fig 6:**
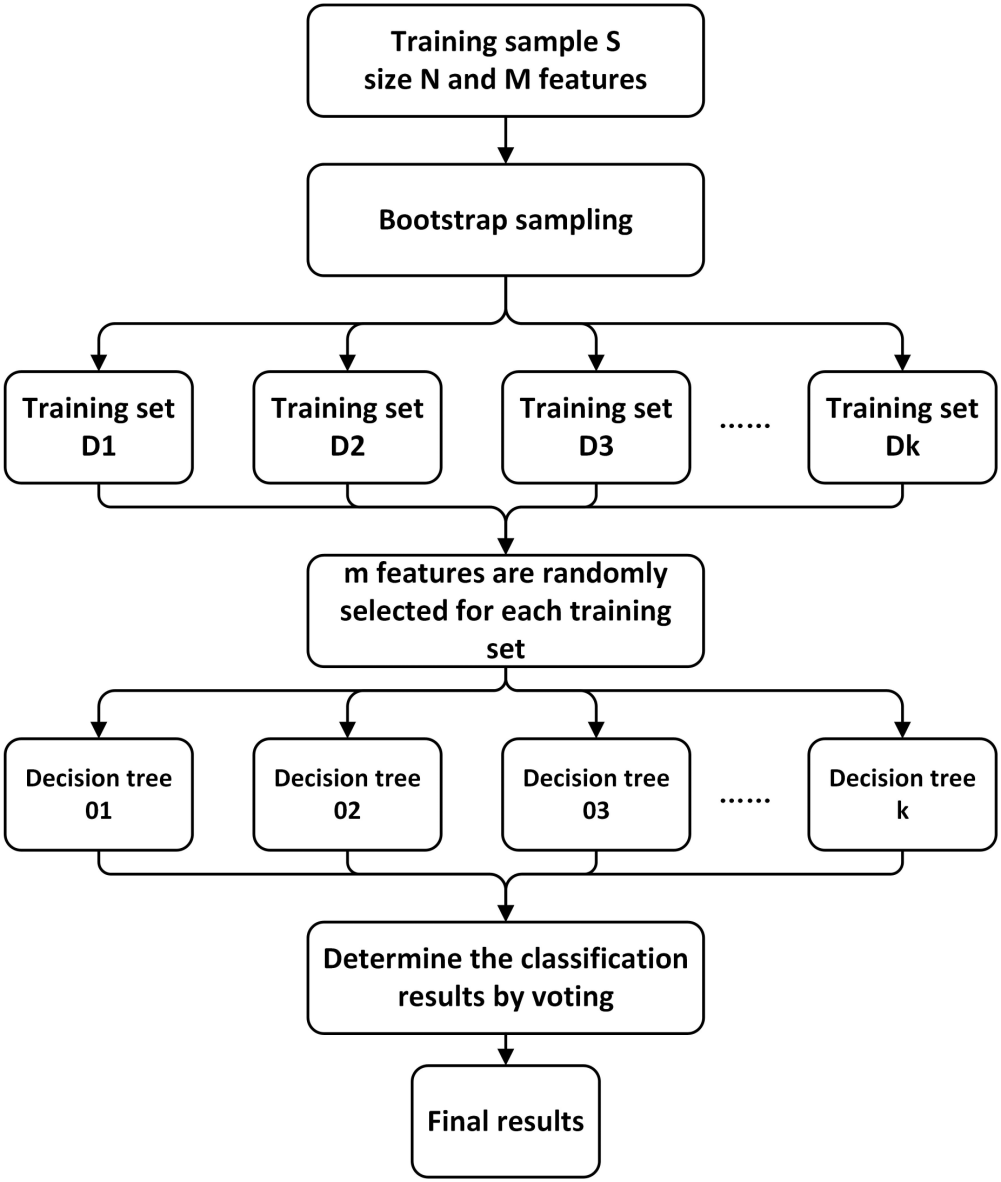
The workflow of the RF algorithm (the size of the training sample*S* is *N*; the sample has *M* features and the forest has *k* trees).

The random forest algorithm was initially proposed by Breiman [97]. It represents a popular tree-based ensemble learning method known for its high adaptability to data features and widespread application in many classification problems [98]. Within the RF model, each individual decision tree is employed for classification, and the ultimate classification outcome is determined by the collective voting of several decision trees. There are several advantages associated with the use of RF model in this research. Firstly, the model demonstrates robust anti-overfitting capabilities and maintains stability due to the incorporation of two random processes. Secondly, the RF model excels in handling discrete data, where the facial data and fatigue data been collected in this case study are high-dimensional and discrete. The workflow of the RF algorithm is visually depicted in Fig 6, and further details can be found in Table 2.

**Table 2:** The steps of applying RF algorithm.

Step sequence	Description
**Step 1:**	For each decision tree, sampling N times randomly from the training sample S with replacement (bootstrap sampling method) to form the training subset D.
**Step 2:**	A decision tree is constructed by randomly selecting m features (m<M). Each node of the tree is bifurcated according to certain rules.
**Step 3:**	Repeat Steps 1 and 2 k times to build k decision trees. Each individual decision tree may produce different classification results due to the selection of different sample subsets and features.
**Step 4:**	The RF model is constructed by all the decision trees.

## 4. Pilot study

### 4.1. Data collection of pilot study

A pilot study is applied here to validate the efficiency of the proposing method. The study involved 10 operators (students) as participants. The participant group consisted of 5 female operators (50%) and 5 male operators (50%). This research simulates the working environment of control room operators within the chemical industry. The specific task assigned to participants involves observing and recording abnormal data presented on the interface of the Distributed Control System (DCS) platform. In daily work, the operation process is significantly complex. However, we designed the experiment tasks to be simple and monotonous, thereby facilitating a quicker onset of fatigue among participants, which aids in the data collection process. The experiments are also conducted after lunchtime because it is conducive to gathering data on human fatigue.

**Fig 7:**
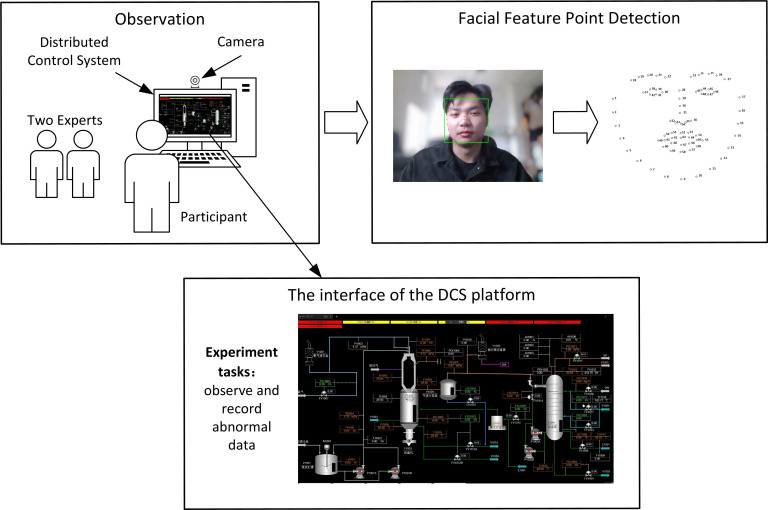
The overall workflow of the experiment.

During the experiment, participants engaged in a 30-minute operation of the DCS. A camera captured photos of the participants at 2-second intervals, and the Landmarks algorithm was applied to extract facial feature points from these photos. There are totally 7940 valid face images in dataset. These images were subsequently randomized and evaluated by two experts employing a modified version of the KSS scale specific to this research. Additionally, participants provided their own self-assessments. The final score was determined by calculating the average of the three results. The overall experimental setup is outlined in Fig 7. The participants’ mental fatigue status was firstly assessed using the KSS measurement. In the traditional KSS, the fatigue state is divided into 10 levels ranging from 1 to 10, see Table 3. However, to avoid potentially misleading the model’s decision-making process, a simplified KSS scale (ranging from 1 to 5 with a step size of 1) was employed in this survey, see Table 4. Ethical approval for this research was obtained from Zhejiang University, the Committee of College of Biomedical Engineering and Instrument Science, under reference number [2022]-No.48.

**Table 3:** The original Karolinska Sleepiness Scale.

Fatigue description	Fatigue level
Extremely alert	1
Very alert	2
Alert	3
Rather alert	4
Neither alert nor sleepy	5
Some signs of sleepiness	6
Sleepy, but no effort to keep awake	7
Sleepy and some effort to keep awake	8
Very sleepy, great effort to keep awake, fighting sleep	9
Extremely sleepy, can’t keep awake	10

**Table 4:** The modified KSS and experiment sample size.

Fatigue description	Fatigue level	Description	Sample size in our experiment
Very alert	1	No sign of drowsiness at all.	278
Relatively alert	2	Alert, slightly less than state “Very alert”.	1305
Some signs of sleepiness	3	Less drowsiness.	3463
Sleepy	4	Need to try to stay awake.	2497
Extremely sleepy	5	Can barely or can’t stay wake.	396

### 4.2. Data processing of the experiment

**Fig 8:**
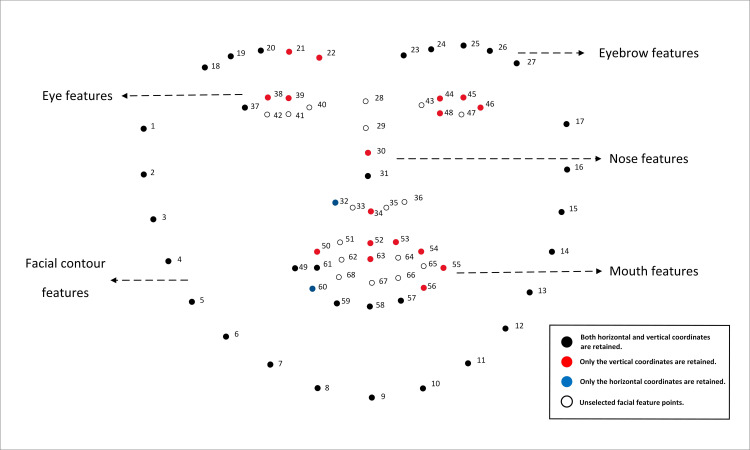
A total of 83 selected features.

Firstly, the facial data undergoes outlier processing. Values that fall beyond three standard deviations above or below the mean are classified as outliers. Such outliers are subsequently removed from the dataset and replaced with the mean of the preceding and succeeding values. This is necessary because participants may exhibit facial occlusions during the experiment, which can result in incomplete facial data. Following this, to avoid variations among participants, a standardization process is applied to mitigate differences. After standardization, the absolute positional relationship of the coordinates was converted into a relative positional relationship, ensuring that the detection process remained unaffected by the participants’ positions within the photographs. Subsequently, the feature selection step is initiated. The RFECV method is used to eliminate the interference of redundant features on MFD, with RF been chosen as the estimator of RFECV. To address the effects of randomness, RFECV is combined with Monte Carlo simulation in this research, with the RFECV process repeated 100 times. For each feature, when the selection frequency exceeds a certain threshold, the feature is retained. Conversely, if the frequency falls below this threshold, the feature is eliminated. The range of the decision threshold spans from 0 to 1. The closer threshold is to 1, the higher the result’s stability, but there is a risk of overlooking important features. Conversely, the closer threshold is to 0, the result becomes more complex and retains more features, but this leads to a significant increase in the computational cost of model training and testing. In this survey, the value of the decision threshold is determined by the following two reasons. Firstly, one of the research aims is to look for more potential features that may exist related to fatigue, fewer retained features may hide this potential relationship. Secondly, cross-validation techniques are used for threshold selection and the results are shown in the Table 5. The result shows that in the range from 0 to 1, threshold 0.1 has the highest cross- validation score, which means it is the best threshold. Therefore, in this work, threshold is chosen to be 0.1. A total of 83 features are ultimately selected, as detailed in Table 6. The selected mental fatigue features are visualized in Fig 8, with different representations for various types of features, hence black dots represent feature points where both horizontal and vertical coordinates are retained, blue dots represent feature points where only the horizontal coordinates are retained, red dots represent feature points where only the vertical coordinates are retained, and white dots represent neither horizontal nor vertical coordinates are retained.

**Table 5:** Cross- validation result in threshold selection.

Threshold	Cross-validation Score
0.1	0.7687
0.25	0.7593
0.5	0.7680
0.75	0.7613
0.9	0.7593

**Table 6:** MFD related features selected by using RFECV. (the ‘~’ represents that all features in the interval are retained; x represents horizontal coordinate, y presents vertical coordinate).

Class	Feature
Facial contour features	x1 ~ x17, y1 ~ y17
Eyebrow features	x18, x19, x20, x23 ~ x27, y18 ~ y27
Nose features	x31, x32, y30, y31, y34
Eye features	x37, y37, y38, y39, y48, y44, y45, y46
Mouth features	x49, x57 ~ x61, y49, y50, y52 ~ y59, y61, y63

### 4.3. Mental fatigue detection output of the experiment

The primary purpose of developing the mental fatigue detection model is to classify and differentiate fatigue status. Hence, existing methods for fatigue status classification are used to compare with the DRN-RF model to examine its accuracy and stability. The examined methods include ANN, GBM, KNN, and RF.

The parameters of GBM, KNN and RF are determined by the GridSearchCV method, see Tables 7–9. The parameters of the DRN-RF are determined as follows. In this research, the strategy for tuning hyperparameters within the proposed DRN model is as follows. Firstly, the GridSearchCV method is employed to determine the range of hyperparameters. This involves systematically exploring different possible ranges for parameters such as the number of layers in the network, the number of neurons in each hidden layer, as well as the values for ‘epochs’ and ‘batch_size’. Through an extensive exploration of these ranges, then conduct iterative trials to gradually refine the parameter combinations to achieve the optimal state. Experimental results have demonstrated that for the DRN, the highest accuracy is achieved under a specific configuration. Specifically, when the network consists of 15 layers and each hidden layer contains 32 neurons, while the parameters ‘epochs’ and ‘batch_size’ are set to 100 and 32 respectively. The setting of ‘epochs’ to 100 implies that the entire dataset is trained 100 times in total during the training process. Meanwhile, the ‘batch_size’ being set to 32 indicates that each individual training batch is composed of 32 samples. This particular combination of hyperparameters has been verified to yield the best performance in our experiments on the DRN model.

**Table 7:** Parameters of GBM.

Parameters	Value
learning_rate	5
max_depth	None
min_samples_leaf	1
min_samples_split	2
n_estimators	300

**Table 8:** Parameters of KNN.

Parameters	Value
n_neighbors	3
metric	manhattan

**Table 9:** Parameters of RF.

Parameters	Value
max_depth	None
max_leaf_nodes	None
min_samples_leaf	1
min_samples_split	2
n_estimators	100

The entire dataset is then partitioned into three sub-datasets, hence a training sub-dataset (comprising 70% of the total dataset), a validation sub-dataset (constituting 15% of the total dataset), and a testing sub-dataset (also accounting for 15% of the total dataset). This division is implemented to mitigate overfitting. Through the introduction of the validation sub-dataset, an effective means is provided to monitor the model’s performance during the training process. By evaluating the model’s performance metrics on the validation sub-dataset at regular intervals, early signs of overfitting can be detected promptly. Subsequently, appropriate adjustments to the training process, such as modifying hyperparameters or implementing early stopping strategies, can be made based on the feedback from the validation sub-dataset, thereby effectively dealing with the overfitting problem and enhancing the model’s generalization ability.

Table 10 indicates the performance of all these methods in MFD, and the results represent the mean of 10 trials. The performance of the models is assessed based on deviation and classification accuracy. The results indicate that DRN-RF attains the highest classification accuracy, reaching 94.2%. Significantly, this accuracy is 17.5% higher compared to that of the RF model. With regard to deviation, the DRN-RF model exhibits a remarkably low deviation of merely 0.004, which is 0.021 less than that of the RF model, which is the best-performing one among the other models under consideration.

**Table 10:** The test results of different MFD comparison.

Method	DRN-RF	ANN	GBM	KNN	RF
**Accuracy**	**94.2%**	58.3%	64.8%	74.8%	76.7%
**Deviation**	**0.004**	0.079	0.108	-0.040	0.025

To test for performance differences between the proposed method and other widely used approaches, the Analysis of Variance (ANOVA) method is employed. A significance level of 5% is applied to all tests, see results in Table 11, reveal that when p <  0.05, it signifies a significant difference between the compared algorithms. A higher f-value indicates a more significant difference. The results demonstrate that, when considering the p-values related to classification accuracy and deviation, the p-values obtained by comparing the DRN-RF model with other models are substantially less than 0.05. This statistical finding clearly implies that there exist significant differences between the DRN-RF model and the other models under investigation. From the perspective of the f-value, it is observed that the f-values corresponding to both classification accuracy and deviation are significantly greater than 1. Notably, the f-value of accuracy reaches as high as 1.94 × 10⁴. Such high f-values further emphasize that the differences between the DRN-RF model and the other models are highly significant. Moreover, in conjunction with the data presented in Table 10, it can be comprehensively concluded that the DRN-RF model exhibits a remarkable superiority over other methods in terms of both accuracy and stability.

**Table 11:** Comparison of difference between DRN-RF and the others.

Algorithm	Algorithm	Accuracy improvement	p_val. (Accuracy)	f_val (Accuracy)	Deviation improvement	p_val. (Deviation)	f_val. (Deviation)
**DRN-RF**	**ANN**	35.9%	5.17×10-28	1.61×104	0.075	7.37×10-3	9.12
	**GBM**	29.4%	9.39×10-29	1.94×104	0.104	3.83×10-17	9.80×10^2^
	**KNN**	19.4%	3.79×10-20	2.13×103	0.044	3.08×10-7	61.98
	**RF**	17.5%	1.27×10-26	1.12×104	0.021	8.70×10-7	53.38

This research also considers the model performance on sleepy and extremely sleepy detection separately, because the detection of these two states is the most important part of mental fatigue detection. The results of comparison and ANOVA is shown in Table 12 and Table 13. The results also confirm that the proposed DRN-RF method exhibits significantly superior stability and accuracy compared to the other approaches in Sleepy and Extremely Sleepy examination.

**Table 12:** The test results of different MFD comparison in Sleepy & Extremely Sleepy.

Method	DRN-RF	ANN	GBM	KNN	RF
**Accuracy**	**94.3%**	56.6%	64.4%	73.0%	74.1%
**Deviation**	**0.067**	0.485	0.398	0.375	0.304

**Table 13:** Comparison of difference between DRN-RF and the others in Sleepy & Extremely Sleepy.

Algorithm	Algorithm	Accuracy improvement	p_val. (Accuracy)	f_val (Accuracy)	Deviation improvement	p_val. (Deviation)	f_val. (Deviation)
**DRN-RF**	**ANN**	37.7%	3.50×10-12	2.63×102	0.418	1.49×10-11	2.21×10^2^
	**GBM**	29.9%	2.56×10-20	2.23×103	0.331	2.51×10-18	1.33×10^3^
	**KNN**	21.3%	1.06×10-23	5.32×103	0.308	4.36×10-23	4.54×10^3^
	**RF**	20.2%	1.48×10-22	3.96×103	0.237	3.02×10-20	2.19×10^3^

**Fig 9:**
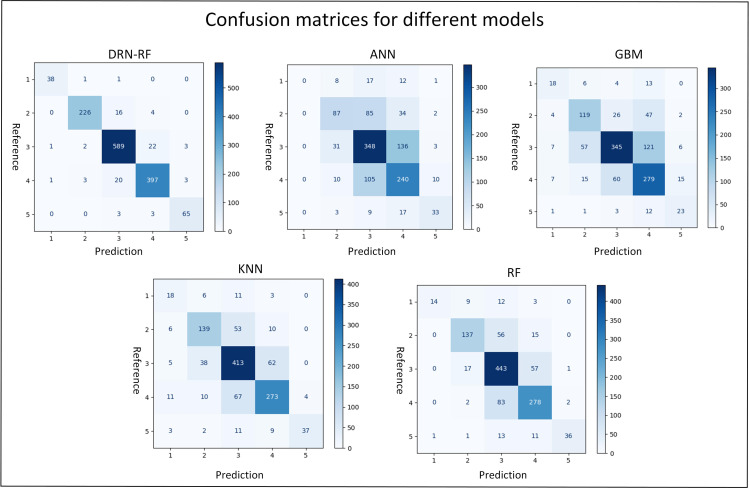
The confusion matrices of different MFD models comparison.

Simultaneously, by referring to the confusion matrix presented in Fig 9, the classification performance regarding “Sleepy” and “Extremely Sleepy” in each model is conducted, with the results shown in Table 14. Here, the Accuracy, Precision, Recall, and Sensitive are chosen as the performance indicators. In Fig 9, the horizontal axis “Prediction” represents the distribution of the model prediction results on each fatigue category, and the vertical axis “Reference” represents the true distribution on each category. It should be noted that the values of these four indicators fall within the range of 0–1, and a larger value indicates a better classification performance of the model in the corresponding category. The data presented in the table further corroborates the conclusion that the DRN-RF model outperforms other models.

**Table 14:** The performance of different MFD models comparison in Sleepy & Extremely Sleepy.

	Sleepy					Extremely Sleepy				
	DRN-RF	ANN	GBM	KNN	RF	DRN-RF	ANN	GBM	KNN	RF
**Accuracy**	**0.96**	0.723	0.757	0.852	0.854	**0.991**	0.951	0.965	0.965	0.976
**Precision**	**0.932**	0.547	0.591	0.764	0.764	**0.915**	0.448	0.5	0.902	0.923
**Recall**	**0.936**	0.658	0.742	0.748	0.762	**0.915**	0.31	0.575	0.596	0.581
**Sensitive**	**0.936**	0.658	0.742	0.748	0.762	**0.915**	0.31	0.575	0.596	0.581

According to the above findings, the study confirms the influence of mouth features, eye features, and nose features on mental fatigue, aligning with the existing studies. It also highlights the significance of eyebrow features and contour features in reflecting human fatigue, consistent with [84], which are often ignored in the previous computer vision based mental fatigue detection.

**Fig 10:**
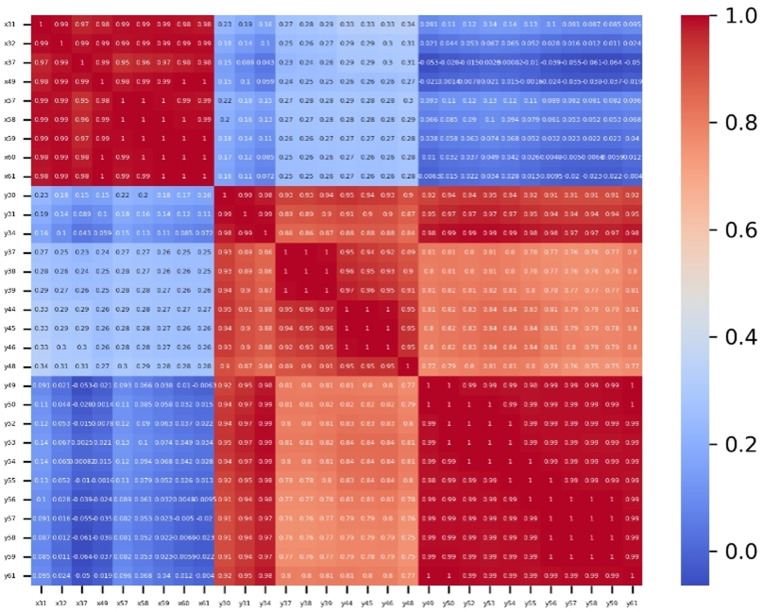
Characteristic correlation thermal diagram. (Feature correlation coefficients among three classes, including eye features, nose features, and mouth features. Red color represents strong correlation, and blue color represents poor correlation).

The Pearson correlation coefficient thermal diagram is shown in Fig 10, where the blue color indicates low coefficient, and the red color indicates high coefficient. The characteristic correlation heatmap reveals those features representing horizontal coordinates are highly correlated with each other, and features representing vertical coordinates are also highly correlated with each other, even when these features do not belong to the same class. For example, feature x31, x32 (nose features), and feature x60, x61 (mouth features), although these features do not belong to the same facial region, they are highly correlated and all contribute to mental fatigue. We also found that even the features belong to same facial region, the horizontal and vertical features are poorly correlated, for example, the eye feature x37 and y37. The above implies that fatigue detection should not be developed solely based on specific facial parts, such as eyes (PERCLOS) and mouth (yawn), because features from different facial regions may be interconnected, and collectively reflecting the mental fatigue state.

## 5. Discussion and conclusion

### 5.1. Contribution of the work

This study has several contributions. Firstly, a new solid method for CRO’s MFD is introduced, employing a combination of computer vision and machine learning referred to as DRN-RF. DRN-RF delivers a potential contribution to enhancing the safety of process system operations, particularly in the area of human factor management. The proposed method is also validated through a pilot study. Secondly, comparing to other existing mental fatigue assessment methods, the proposed method relies on participants’ facial data, which simplifies data collection and enhances the feasibility for daily application in a real working environment. Furthermore, to mitigate the errors introduced by randomness during the feature selection, we integrate the RFECV feature selection method with Monte Carlo theory. This is different from traditional RFECV feature selection, and the combination enhances the reliability of the feature selection process. Alternatively, the stacking model DRN-RF within the proposed approach demonstrates superior detection accuracy and lower deviation compared to other widely used detection models, such as ANN, GBM, and RF. A comparison also highlight that the stability of DRN-RF significantly outperforms the other approaches. The DRN-RF model is developed through ensemble learning, effectively amalgamating the strengths of both DRN and RF. This proposed DRN-RF model not only inherits DRN’s robust feature representation capabilities, which proficiently tackle deep learning’s degradation issues, but also demonstrates outstanding performance in handling high-dimensional and discrete data, as exemplified by the RF model. Given the notable superiority of the DRN-RF algorithm, it is reasonable to consider that many other industries such as vessel operation and vehicle driving could be served as potential application. Nevertheless, in light of the distinctiveness inherent in different scenarios and specific cases, these presumptions still require further in-depth investigation and empirical verification to establish their validity and feasibility within the academic and practical domains. Lastly, in addition to acknowledging the impact of eye features and mouth features on mental fatigue, as considered in previous studies, this research identifies that facial contour features and eyebrow features also play important roles in individual’s mental fatigue. This discovery has the potential to enrich the feature selection process in future MFD studies.

### 5.2. Limitation and future work opportunity

This study contributes to mental fatigue detection by analyzing facial feature data. However, it is important to acknowledge certain limitations associated with the establishment of the human facial dataset. One limitation of this study concerns the reliance on KSS scores as labels for the dataset. These scores are derived from evaluations by a panel consisting of two domain experts and the participants themselves, which introduces an element of subjectivity into the assessment of fatigue states. Future research could aim to reduce this subjectivity by incorporating more objective measures, such as PERCLOS or other physiological indicators, to serve as the basis for evaluating fatigue levels. Additionally, alternative metrics may be employed to monitor and validate the accuracy of the labeling process. Another limitation of this study arises from the occurrence of facial occlusion during the data collection process, which can be attributed to variations in lighting conditions and the potential for unreliable image capture, thereby affecting the completeness of the dataset. Given that our research focuses on control room operators, the influence of illumination on image quality can be mitigated by controlling the indoor light sources. In cases of occlusion, we will implement a processing method, which is detailed in the section 4.2. To further address this issue, subsequent research aimed at enhancing the Landmark algorithm may be beneficial in reducing the effects of facial occlusion. Another practical limitation is that the method requires the operator to be facing the camera, i.e., in a stationary work position. Industrial control room environments do not always provide this level of postural control. On the other hand, driving tasks do. Consequently, deployment of the method is expected to be context-specific. Note also that the present work compared a variety of AI computational approaches applied to facial data. It did not include biological signals, so an analysis of comparative performance is unavailable.

One of the opportunities to the future work is associated with harmonizing other industrial applicable features, e.g., eye features with facial data for mental fatigue assessment. For example, the relationship between eye movement data, such as pupil diameter, and mental fatigue is acknowledged; however, it is imperative to recognize that various external factors can also induce changes in pupil diameter, such as lighting. Hence, a more comprehensive research approach is suggested. Another opportunity of the research is associated with real industrial applications. In a real working environment, the way to collect facial fatigue data might vary due to differences in work content and workload. Hence, the approach to achieving real-time applications and the performance of the DRN-RF model with real data needs further discussion. Nevertheless, in the pilot study, the DRN-RF model stood out over other traditional MFD methods, such as ANN.

### 5.3. Conclusions

Mental fatigue assessment plays an important role in analyzing the reliability of CROs. Most existing MFD methods need multi-dimensional data for effective fatigue detection, but getting such data in daily work environments is very challenging. In this study, a new MFD method combining computer vision and machine learning is proposed. Unlike other methods, it only requires collecting human facial data, making it possible to be applied in daily work environments. The results show that a stacking model DRN-RF performs well when detecting the mental fatigue and is significantly superior to other methods in terms of stability, accuracy and generalization ability.

## Supporting information

S1 Dataset Mental fatigue dataset. (ZIP)
